# Accuracy and Reproducibility of Voxel Based Superimposition of Cone Beam Computed Tomography Models on the Anterior Cranial Base and the Zygomatic Arches

**DOI:** 10.1371/journal.pone.0016520

**Published:** 2011-02-09

**Authors:** Rania M. Nada, Thomas J. J. Maal, K. Hero Breuning, Stefaan J. Bergé, Yehya A. Mostafa, Anne Marie Kuijpers-Jagtman

**Affiliations:** 1 Department of Orthodontics and Oral Biology, Radboud University Nijmegen Medical Centre, Nijmegen, The Netherlands; 2 3D Facial Imaging Research Group Nijmegen – Bruges, Radboud University Nijmegen Medical Centre, Nijmegen, The Netherlands; 3 Department of Oral and Maxillofacial Surgery, Radboud University Nijmegen Medical Centre, Nijmegen, The Netherlands; 4 Department of Orthodontics, Faculty of Oral and Dental Medicine, Cairo University, Cairo, Egypt; Stanford University Medical Center, United States of America

## Abstract

Superimposition of serial Cone Beam Computed Tomography (CBCT) scans has become a valuable tool for three dimensional (3D) assessment of treatment effects and stability. Voxel based image registration is a newly developed semi-automated technique for superimposition and comparison of two CBCT scans. The accuracy and reproducibility of CBCT superimposition on the anterior cranial base or the zygomatic arches using voxel based image registration was tested in this study. 16 pairs of 3D CBCT models were constructed from pre and post treatment CBCT scans of 16 adult dysgnathic patients. Each pair was registered on the anterior cranial base three times and on the left zygomatic arch twice. Following each superimposition, the mean absolute distances between the 2 models were calculated at 4 regions: anterior cranial base, forehead, left and right zygomatic arches. The mean distances between the models ranged from 0.2 to 0.37 mm (SD 0.08–0.16) for the anterior cranial base registration and from 0.2 to 0.45 mm (SD 0.09–0.27) for the zygomatic arch registration. The mean differences between the two registration zones ranged between 0.12 to 0.19 mm at the 4 regions. Voxel based image registration on both zones could be considered as an accurate and a reproducible method for CBCT superimposition. The left zygomatic arch could be used as a stable structure for the superimposition of smaller field of view CBCT scans where the anterior cranial base is not visible.

## Introduction

Three-dimensional digital records are becoming more and more popular among orthodontists and maxillofacial surgeons as the specialties progress towards a three dimensional (3D) virtual representation of the patient for diagnosis, treatment planning and simulation. Cone Beam Computed Tomography (CBCT) scans have been well established as a valuable tool in the orthodontist's and surgeon's 3D toolkit. A single scan not only provides an overlap-free 3D visualization of the skull but also allows detailed evaluation of the maxillofacial structures in thin axial, coronal and sagittal slices. Superimposition of serial cephalometric radiographs has been traditionally used for assessment of growth and treatment effects or stability over a certain time interval. Nowadays, superimposition of CBCT scans allows a three dimensional visualization of these effects. Similar to cephalometric tracings, 3D models constructed from CBCT scans could be superimposed manually by registering common stable landmarks or by best fit of stable anatomical regions [1], [2]. These two methods however depend on the accuracy of landmark definition and the precision of the 3D surface models. Voxel-based image registration is a recently developed automated registration technique whereby CBCT scans are superimposed by comparing the grey values in a defined volume of interest in two scans to compute the rotation and translation required to align the two datasets [3], [4], [5].

Using voxel based image registration, Cevidanes *et al.* [6], [7] described the superimposition of CBCT scans on the anterior cranial base structures for both growing and non growing subjects. They assessed alterations in the 3D position of the mandibular rami and condyles in patients receiving orthognathic surgery. While they demonstrated the reproducibility of this method for CBCT superimposition in the assessment of treatment changes, the accuracy of the superimposition procedure itself at the anterior cranial base was not reported in their studies. Heymann *et al.* [8] used the same superimposition procedure to determine anatomic changes following maxillary protraction with intermaxillary elastics to miniplates. They concluded that 3D data from CBCT allowed a more thorough documentation of the treatment changes. Another interesting application of voxel based CBCT superimpositions was presented by Swennen *et al.* [4]. They used triple voxel-based rigid registration to built an augmented 3D skull model with detailed occlusal and intercuspation data without the use of plaster dental models.

Despite the growing application of CBCT superimposition to assess changes between serial CBCT scans, neither the accuracy of CBCT scans superimposition techniques nor the choice of structures for 3D superimposition have been directly investigated yet. The anterior cranial base has been traditionally considered as a stable structure for the superimposition of serial two dimensional radiographs. It could be regarded as a stable structure for CBCT superimposition as well. However, this region is only visible in an extended height CBCT scan. It has been shown that reducing the scan height or the Field of View (FOV) from the larger size to the next available smaller size results in a significant reduction, up to 50%, in the radiation dosage to the patient [9]. Many healthcare providers nowadays advocate the use of smaller field of view scans to achieve a balance between what this new technology has to offer to the clinician and the radiation dosage to the patient. The objectives of this study were therefore to evaluate accuracy and reproducibility of a new semi-automated voxel based image registration technique for the superimposition of 3D CBCT models on two different regions, the anterior cranial base and the zygomatic arches as proposed new region for CBCT superimposition in smaller field of view scans.

## Materials and Methods

The material for this study consisted of pairs of CBCT scans of 16 adult patients (26±9 yr) retrieved from the Radboud University Nijmegen Medical Centre CBCT database of patients who underwent combined surgical orthodontic treatment. Inclusion criteria were a severe maxillary transverse deficiencies combined with class II or class II malocclusion or open bite, which required two orthognathic surgical interventions. The first CBCT scan was taken prior to treatment while the second was taken before the second orthognathic surgery, on average 18 (±4.6) months later. The study protocol was approved by the Medical Ethical Commission of the Radboud University Nijmegen Medical Centre, Nijmegen, The Netherlands (181/2005). All patients signed the informed consent. The scans were acquired using the i-CAT® 3D Imaging System (Imaging Sciences International Inc, Hatfield, PA, USA) with a field of view of 22×16 cm and 0.4 mm voxel size. Data from the CBCT were exported in Digital Imaging and Communications in Medicine (DICOM) format to Maxilim software (Medicim, Mechelen, Belgium).

### Superimpositions

3D models were constructed and superimposed using voxel based superimposition in Maxilim software installed on a windows XP-based workstation (Intel® core™ 2 Duo; 2.9 GHz, 3.25GB, ATI Radeon™ 3450 HD graphics card). The construction of the 3D models was performed by selecting the range of Hounsfield unit (HU) representing the bony tissues on the DICOM images. This was achieved by selecting a lower threshold value between 250–350 HU. Values above this threshold were automatically selected. The superimposition procedure is an automated procedure that compares the grey values in the two DICOM images voxel by voxel. The user is first required to select the volume of interest (registration area), then to roughly align the 3D models. Consequently the software computes the translation and rotation needed to geometrically align the two DICOM images, and subsequently the constructed 3D models, based on the maximization of mutual information. For each pair of CBCT scans the 3D model construction and superimposition procedure was repeated five times with a time interval of three weeks.

**Figure 1:**
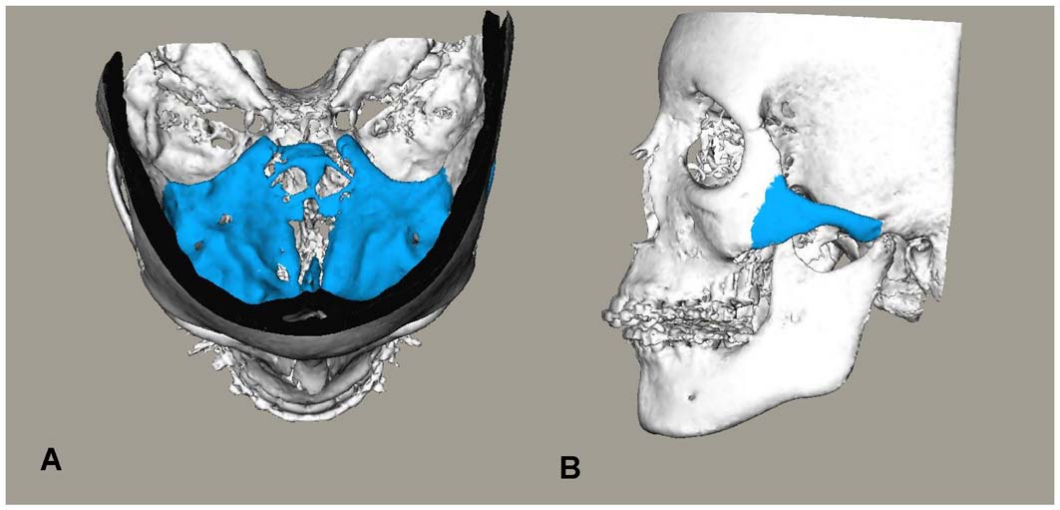
Anatomic structures used for registration highlighted on 3D CBCT models. Anatomic structures used for the registration highlighted on the 3D CBCT models. (A) Anterior cranial base. (B) left zygomatic arch.

The scans were registered twice on the anterior cranial base and twice on the left zygomatic arch (zygomatic bone + zygomatic process of the temporal bone) by the same operator (RN) (Fig. 1). To test the inter-observer reliability, the scans were superimposed for a fifth time by a second observer (HB) registered on the anterior cranial base.

### Testing the Accuracy of the Superimpositions

**Figure 2:**
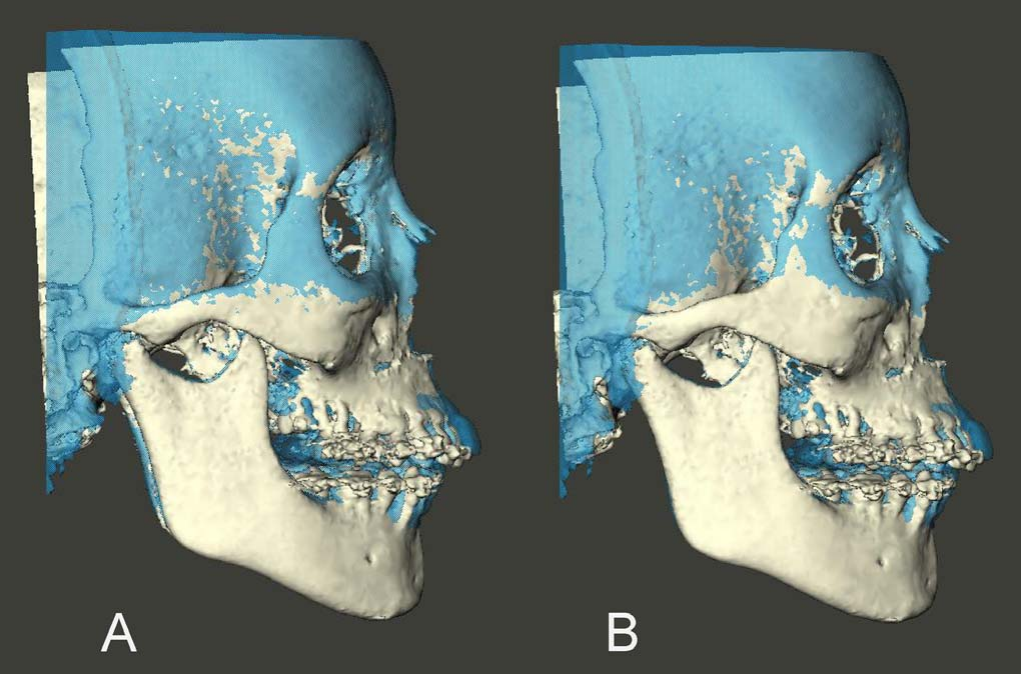
Transparency overlay of superimposed 3D CBCT models. Right side view. (A) models registered on the anterior cranial base. (B) same models registered on the left zygomatic arch.

**Figure 3:**
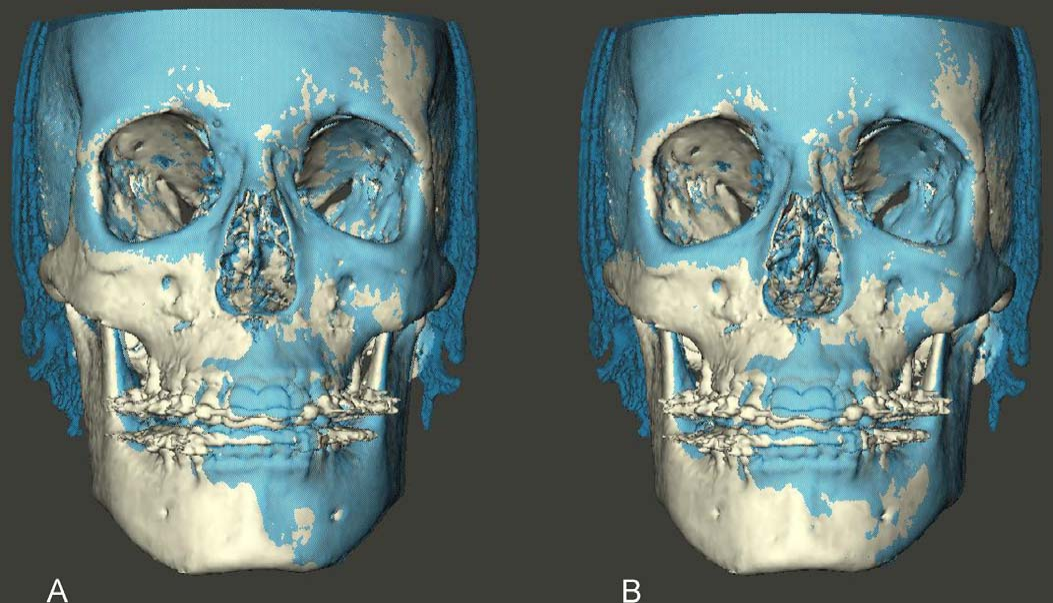
Transparency overlay of superimposed 3D CBCT models. Frontal view. (A) models registered on the anterior cranial base. (B) same models registered on the left zygomatic arch.

**Figure 4:**
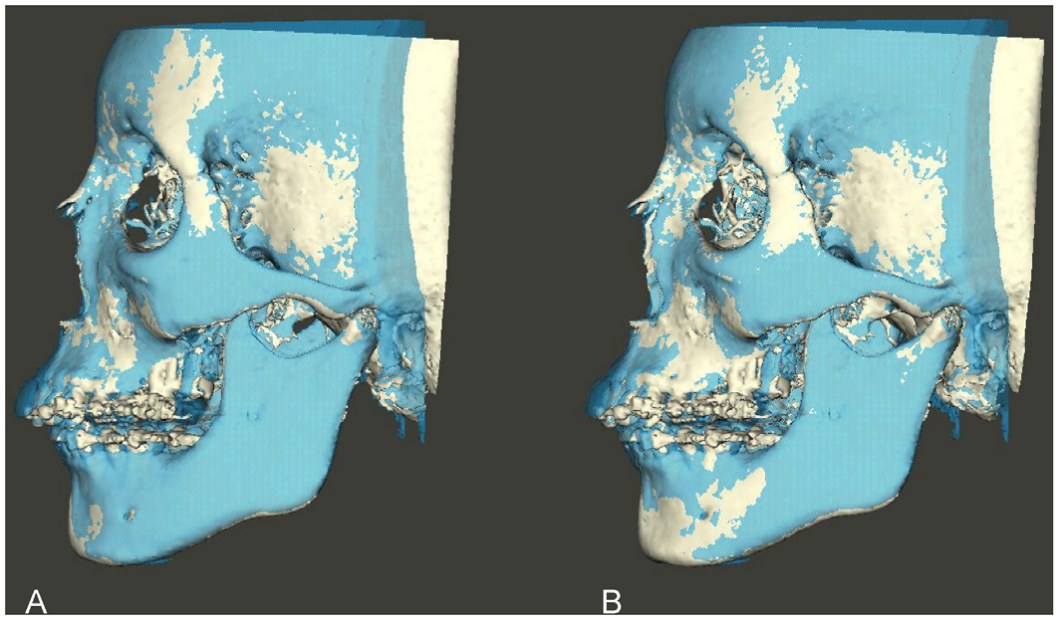
Transparency overlay of superimposed 3D CBCT models. Left side view. (A) models registered on the anterior cranial base. (B) same models registered on the left zygomatic arch.

**Figure 5:**
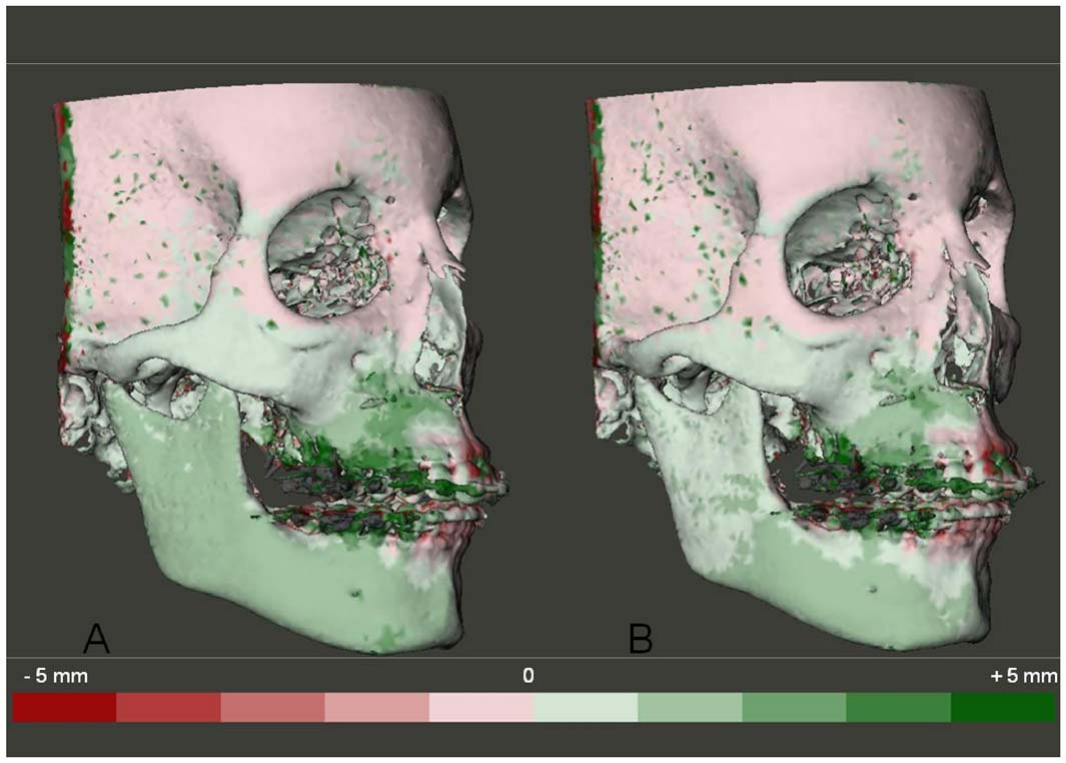
Color coded distance maps to visualize treatment changes following two CBCT scans superimposition. The green color indicates that the superimposed model is in front of the original model and red color indicates the opposite. Each color graduation is 1 mm. (A) models registered on the anterior cranial base. (B) same models registered on the left zygomatic arch.

**Figure 6:**
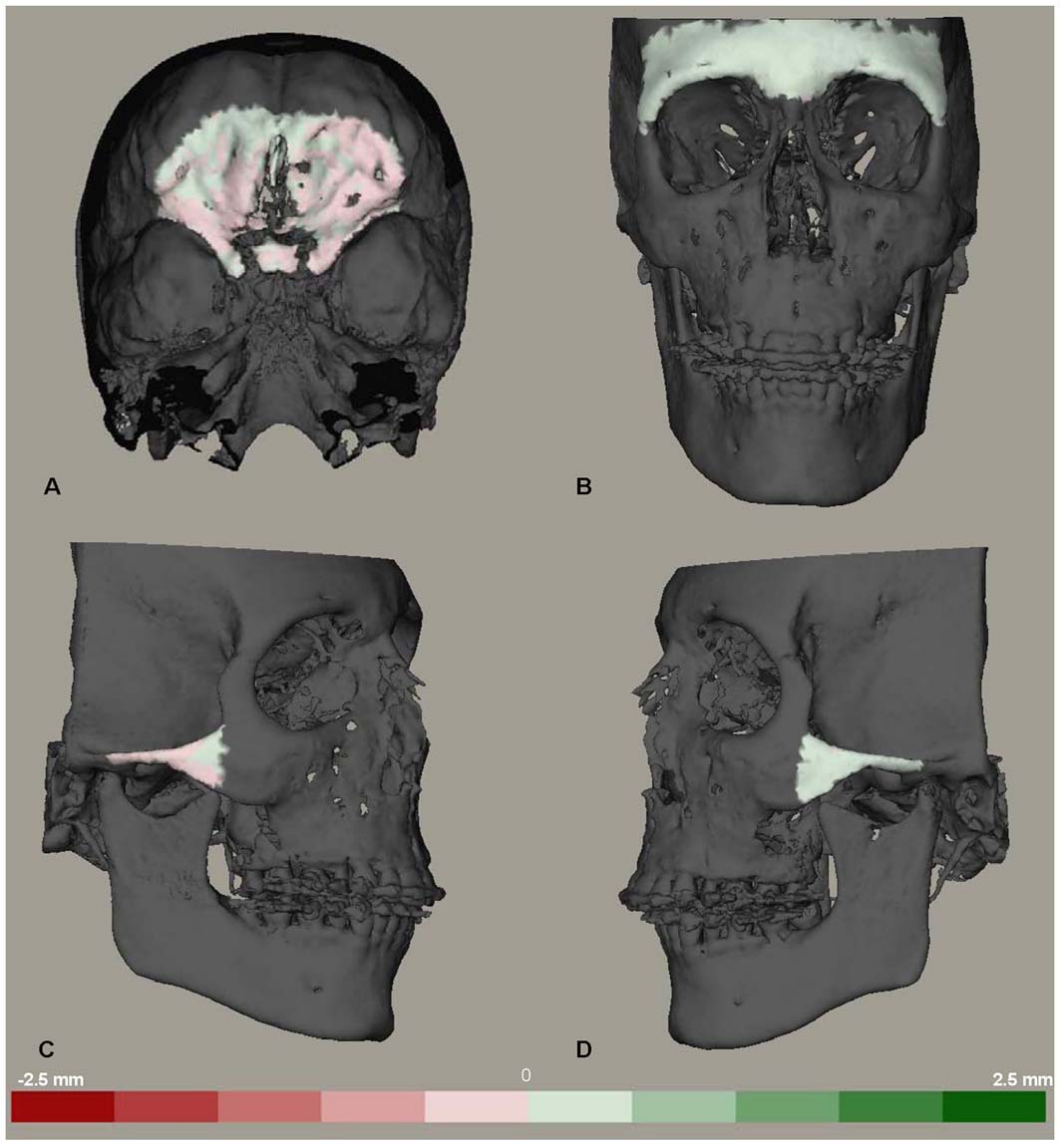
Distance maps to visualize the distances between two models registered on the anterior cranial base. Color coded distance maps to visualize the distances between two superimposed models registered on the anterior cranial base. The green color indicates that the superimposed model is in front of the original model and red color indicates the opposite. Each color graduation is 0.5 mm. (A) anterior cranial base. (B) the forehead region. (C) the right zygomatic arch. (D) the left zygomatic arch.

**Figure 7:**
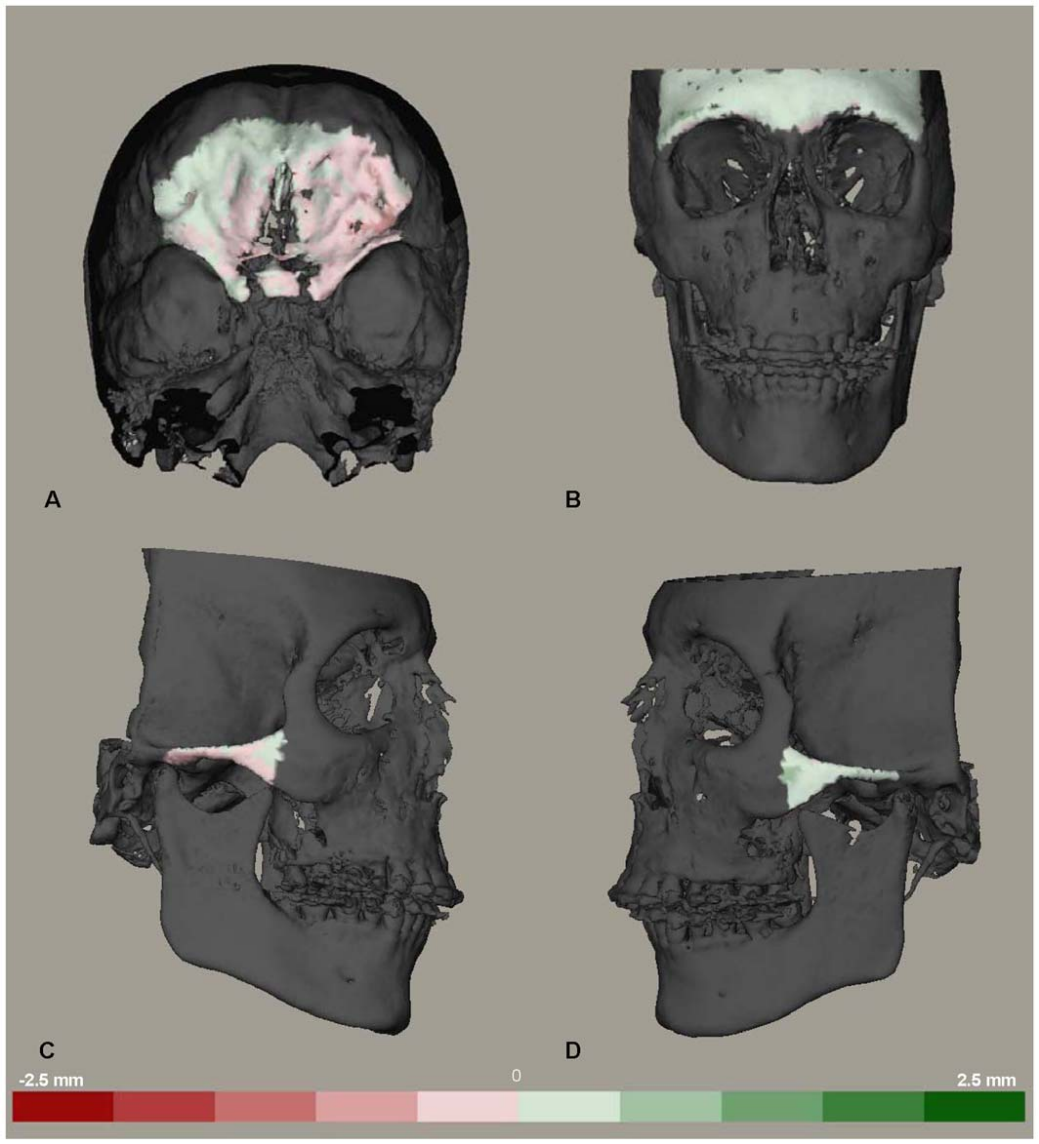
Distance maps to visualize the distances between two models registered on the left zygomatic arch. Color coded distance maps to visualize the distances between two superimposed models registered on the left zygomatic arch. The green color indicates that the superimposed model is in front of the original model and red color indicates the opposite. Each color graduation is 0.5 mm. (A) anterior cranial base. (B) the forehead region. (C) the right zygomatic arch. (D) the left zygomatic arch.

Following each superimposition, using Maxilim software, color coded distance maps as well as transparency overlays were constructed to visualize the superimposed models (Fig. 2, 3, 4 and 5). The mean absolute distances between the two 3D models were computed in 4 different regions: the anterior cranial base, the forehead, left and right zygomatic arches (Fig. 6 and 7). The absolute values of the distances were exported to excel sheets and the mean value for each region was calculated.

### Statistical Analysis

The intra-observer and inter-observer reliability was calculated using the Pearson correlation coefficient for the mean distances at the 4 anatomical regions following the first and second superimpositions. Paired-sample *t*-test was performed to compare the means of corresponding measurements following registration on the anterior cranial base and the left zygomatic arch. The significance level was set at 5%.

## Results

The time required to complete a single superimposition procedure ranged from 30 to 40 min. The mean and standard deviation of the mean distances between the superimposed models at the four regions following the five superimpositions is shown in Table 1.

**Table 1:** Mean distances (mm) between the superimposed models measured at 4 different regions following 5 repeated superimpositions.

	Registered on the anterior cranial base									Registered on the zygomatic arch					
Region	S1			S2			S3*			S4			S5		
	mean	SD	SE	mean	SD	SE	mean	SD	SE	mean	SD	SE	mean	SD	SE
CB	0.33	0.12	0.03	0.31	0.07	0.02	0.3	0.12	0.03	0.45	0.22	0.06	0.52	0.35	0.09
FH	0.2	0.08	0.02	0.19	0.08	0.02	0.13	0.03	0.01	0.39	0.22	0.06	0.35	0.16	0.04
ZR	0.3	0.24	0.06	0.37	0.31	0.08	0.34	0.25	0.06	0.45	0.27	0.07	0.44	0.21	0.05
ZL	0.37	0.16	0.05	0.39	0.16	0.04	0.36	0.15	0.04	0.2	0.09	0.02	0.17	0.08	0.02

CB, anterior cranial base; FH, forehead; ZR, right zygomatic arch; ZL, left zygomatic arch; S, superimposition;

*superimposition performed by a second observer.

Table 2 shows the differences between the first and second superimposition on the anterior cranial base. Intra-observer reliability was good between the repeated superimpositions: the correlation coefficients between the first and second superimpositions registered on the anterior cranial base ranged between 0.53 and 0.94 for the mean distances at the 4 regions. The interobserver variability was very small when the 3D models construction and superimposition procedure was repeated by a second observer. Mean differences between the superimpositions performed by the first and second observer were 0.02 mm (SD 0.1) for the anterior cranial base, 0.05 mm (SD 0.05) for the forehead region, −0.04 mm (SD 0.18) for the right zygomatic arch and 0.02 mm (SD 0.14) for the left zygomatic arch.

**Table 2:** Mean differences (mm) and 95% confidence interval (CI) between first and second superimposition registered on the anterior cranial base.

Paired Differencess						
				95% CI of the Difference		
	Mean	SD	SE Mean	Lower	Upper	P-Value
CB.1 - CB.2	0.02	0.09	0.02	−0.03	0.07	0.4
FH.1 - FH.2	0.01	0.07	0.02	−0.03	0.05	0.74
ZR.1 - ZR.2	−0.07	0.12	0.03	−0.13	−0.003	0.04
ZL.1 - ZL.2	−0.01	0.15	0.04	−0.09	0.07	0.74

CB, anterior cranial base; FH, forehead; ZR, right zygomatic arch; ZL, left zygomatic arch; 1, first superimposition; 2, second superimposition; SD, standard deviation; SE, standard error.

Table 3 shows the differences between the two superimpositions registered on the zygomatic arches. The correlation coefficients between the first and second superimpositions ranged between 0.24 and 0.71 for the mean distances at the 4 anatomic regions. The distances between the superimposed models registered on the zygomatic arch were slightly higher than the models registered on the anterior cranial base at 3 regions (Table 4). The mean differences were 0.12 mm (SD 0.19) for the anterior cranial base, 0.19 mm (SD 0.12) for the forehead region, and 0.15 mm (SD 0.18) for the right zygomatic arch. On the other hand, the distance between the two models decreased at the left zygomatic arch mean difference was -0.17 mm (SD 0.13). The *P*-values ranged between 0.001 and 0.025 and were statistically significant for the 4 regions.

**Table 3:** Mean differences (mm) and 95% confidence interval (CI) between superimpositions registered on the left zygomatic arch.

Paired Differences						
				95% CI of the Difference		
	Mean	SD	SE Mean	Lower	Upper	P-Value
CB.4 - CB.5	−0.07	0.25	0.06	−0.2	0.06	0.29
FH.4 - FH.5	0.04	0.24	0.06	−0.1	0.18	0.53
ZR.4 - ZR.5	0.14	0.1	0.05	−0.09	0.12	0.78
ZL.4 - ZL.5	0.04	0.09	0.02	−0.01	0.09	0.1

CB, anterior cranial base; FH, forehead; ZR, right zygomatic arch; ZL, left zygomatic arch; 4, fourth superimposition; 5, fifth superimposition; SD, standard deviation; SE, standard error.

**Table 4:** Mean differences (mm) and 95% confidence interval (CI) between superimpositions registered on the left zygomatic arch and superimpositions registered on the anterior cranial base.

Paired Differences						
				95% CI of the Difference		
	Mean	SD	SE Mean	Lower	Upper	P-Value
CB.4 - CB.1	0.12	0.19	0.05	0.017	0.22	0.025
FH.4 - FH.1	0.19	0.12	0.05	0.07	0.3	0.004
ZR.4 - ZR.1	0.15	0.18	0.05	0.05	0.24	0.005
ZL.4 - ZL.1	−0.17	0.13	0.03	−0.24	−0.1	0.001

CB, anterior cranial base; FH, forehead; ZR, right zygomatic arch; ZL, left zygomatic arch; SD, standard deviation; SE, standard error; 4, registered on left zygomatic arch; 1, registered on anterior cranial base.

## Discussion

The aim of this study was to test the accuracy and reproducibility of the voxel based superimposition of CBCT scans registered on two different regions: the anterior cranial base and the left zygomatic arch. The accuracy of the superimpositions was tested by calculating the mean absolute distances between the two models at four different anatomic regions: the anterior cranial base, the forehead, the left and the right zygomatic arches. These four regions could be considered as stable structures following orthognathic surgery. The cranial base region was chosen to test alignment errors in the vertical direction, the forehead region for the antero-posterior direction, while the right and left zygomatic arches were chosen for the transverse direction.

To be suitable for routine application in medical image processing, a superimposition procedure should be precise, efficient and should not require an excessive amount of time. The image-analysis procedures used in this study required 30–40 min per set of 2 CBCT scans. This included construction of 3D models, voxel based superimposition of the models, calculation of the distances between the 3D surfaces and generation of color coded distance maps. To our knowledge this required much less time than the procedures reported in previous studies [10]. When the models were registered on the anterior cranial base, the average distance calculated between the models ranged between 0.2 and 0.37 mm. Moreover, the reproducibility of this method was confirmed by the small differences between the repeated superimpositions on the anterior cranial base. The mean difference between the distances of the first and second superimposition procedures ranged between 0.02 to 0.07 mm at the four anatomic regions. This difference was statistically significant at the right zygomatic arch (*P* = 0.04), but the clinical relevance is negligible because of the very small values.

Cevidanes *et al.* [6] studied the variability between observers in quantification of treatment outcome on color coded distance maps for different anatomic regions on 3D CBCT models registered on the anterior cranial base. They reported an inter-examiner range of measurements across anatomic regions equal or less than 0.5 mm. They concluded that the small inter-observer variability could be accounted to the automation of the voxel based registration procedure and its independence from the precision of the 3D surface models. This would be equally applicable to the very small intra-observer and inter-observer variability observed in our study. The mean difference between the superimpositions performed by the two observers ranged between 0.02 and 0.05 mm for the four anatomical regions. It should be noted however, that since the distance maps are constructed on the 3D surface models they could be dependent on the accuracy of the segmentation or the selection of the bone threshold values of these models. While the segmentation procedure in our study was different from the procedure used by Cevidanes *et al.* [6], the results of both studies showed that the potential source of variation due to segmentation was very small.

The zygomatic arches could be considered as stable structures for non-growing patients undergoing single or double jaw surgery. They are clearly visible and easily isolated as a region of interest in CBCT scans. With the growing concern about the radiation dosage from CBCT scans [11], they could offer an added advantage as they are clearly visible in a scan with smaller field of view (FOV) or reduced scan height (13 cm) compared to the anterior cranial base which requires an extended field of view (22 cm). Ludlow *et al.* [9] and others [12], [13], have shown that smaller FOV examinations are associated with significant radiation dose reductions and less tissue radiation especially to the eyes. For the i-CAT machine used in our study, the use of the 13 cm FOV scan results in 50% reduction of the overall radiation dose when compared to the 22 cm scan [9]. When the registration was performed on the left zygomatic arch, the distances between the two superimposed models were slightly larger at the anterior cranial base, the forehead and the right zygomatic arch but were smaller on the left zygomatic arch when compared to superimpositions registered on the anterior cranial base. The mean difference ranged between 0.12 to 0.19 mm. While these differences were found to be statistically significant they are too small to be considered clinically relevant. The mean distances between the two models registered on the zygomatic arch remained within 0.5 mm accuracy advocated by Hajeer *et al.* [14]. Ideally it would be preferred to register the two models on both the right and left zygomatic arches to increase the accuracy of the superimpositions. However, voxel based superimposition could only be performed on one volume of interest at a time using the commercially available software. Hopefully this would be feasible in the near future.

### Conclusion

Voxel based image registration is an accurate and a reproducible semi-automated technique for superimposition of 3D CBCT models. In non growing subjects, registration of the superimposed models on the zygomatic arches could be considered as an alternative to the anterior cranial base in smaller FOV scans.
